# A Protocol for a Systematic Review and Meta-Analysis of Hospital Readmissions Following Acute Upper Gastrointestinal Bleeding

**DOI:** 10.7759/cureus.19263

**Published:** 2021-11-04

**Authors:** Sandeep Kaur, Cody L Dunne, Lauren Bresee

**Affiliations:** 1 Gastroenterology, Cumming School of Medicine, University of Calgary, Calgary, CAN; 2 Emergency Medicine, University of Calgary, Calgary, CAN; 3 Epidemiology and Public Health, Canadian Agency for Drugs and Technologies in Health, Calgary, CAN

**Keywords:** mortality, protocol, systematic review and meta analysis, 30-day readmission, recurrent gi bleeding

## Abstract

This protocol outlines the planned methodology for a systematic review and meta-analysis. The primary objective of the review is to identify all-cause readmission rates for individuals hospitalized for an upper GI bleed (UGIB). Secondary objectives will include GI bleed-specific readmission rates, mortality (all-cause and GI bleed-specific), readmission diagnosis, and length of stay on readmission visit. High-risk subgroups will also be explored including age, sex, type of GI bleed (e.g., variceal or not), anti-coagulation status, and comorbidity status.

Through this review, the research team aims to describe an important quality indicator, which has implications for both patient safety post-discharge after an UGIB and healthcare resource utilization.

## Introduction

Acute upper gastrointestinal bleeding (AUGIB) has been previously defined as blood loss anywhere between the esophagus and the Ligament of Treitz (at the duodenojejunal flexure). In recent years UGIB has been redefined as bleeding above the ampulla of Vater within reach of upper endoscopy [1,2].

UGIB is one of the most common gastrointestinal emergencies, with an incidence of 50 to 160 per 100, 000 population per year and a 6-10% mortality [3,4]. Variceal and non-variceal bleeding are the two main categories of UGIB [5]. Non-variceal lesions account for 80-90% of UGIB cases. These include gastro-duodenal peptic ulcers (20-50%), gastro-duodenal erosions, erosive esophagitis, Mallory-Weiss tears, as well as other conditions. Variceal bleeding largely is esophageal in nature, but occasionally originates from the stomach [1,6]. Varices are due to increased venous pressures, often from portal hypertension as a result of end-stage liver disease [7].

GI bleeding can be fatal and readmission within 30 days after the first episode of bleeding can pose a significant burden on the healthcare system. However, despite readmission rate being known as an important indicator and used in other morbid conditions, there is currently no summary data on this for upper GI bleeds. The purpose of this study is to systematically review and determine the readmission rate, and mortality for patients treated for an AUGIB.

## Materials and methods

Research question

What is the hospital readmission rate for adults discharged from the hospital following treatment for AUGIB?

Systematic review design

Table 1 outlines the framework for the systematic review utilizing a Population-Exposure-Comparator-Outcomes (PECO) structure and is registered on the International Prospective Register of Systematic Reviews (PROSPERO) [8].

**Table 1:** PECO criteria for inclusion of studies into the systematic review and meta-analysis UGIB: Upper gastrointestinal bleed; PECO: Population-Exposure-Comparator-Outcome

Study Feature	Description
Population	Adult patients (> 18 years of age)
Exposure	Discharged from hospital after treatment for an acute upper GI bleed
Comparator	Not applicable
Outcome(s)	
Primary	Readmission rate to hospital (all-cause)
Secondary	Readmission rate to hospital (due to UGIB), mortality (all-cause, and due to UGIB), length of stay, readmission diagnosis
Design (Study)	Randomized and non-randomized trials, observational studies
Time range	Inception to present day

Search Strategy

The online databases to be queried for the systematic review include MEDLINE (1946 - Present), Embase (1976 - present), Web of Science’s Science Conference Proceedings section (1990 - present), and Cochrane Central Register of Controlled Trials (CENTRAL). Two researchers (CD, SK) will review the cited references of all included articles to identify other relevant studies. 

It was decided to search all databases from inception based on a preliminary search, which revealed relatively few (less than 500) studies without any time limits imposed. Non-human studies will be excluded. All languages will be included. In the case that a foreign language publication makes it to abstract or full-text screening and an English translation is unavailable, a translation service will be used.

Two key search concepts were identified from the research question. The researchers identified these themes and then identified common synonyms and alternative spelling that could be present in the literature. The results of this are in Table 2. Appropriate subject headings and keywords embedded in the respective databases will be used in combination with the search terms to capture any relevant studies.

**Table 2:** Table 2: Key search concepts along with alternative spelling and synonyms AUGIB: acute upper gastrointestinal bleed; UGIB: upper gastrointestinal bleed

Key Concept	Potential Variations
Upper gastrointestinal bleed	GI bleed, Hemorrhage, Haemorrhage, GIB, UGIB, AUGIB, Variceal, Non-variceal
Hospital readmission	Readmission, Readmittance, Revisit, Rehospitalization, Rehospitalisation; All of the above with “Re-” as spelling

It was subsequently chosen by the researchers to not utilize “upper” in the search strategy and limit the results. This was decided as it was felt some articles may address GI bleeds in general and may stratify data by type within their results. Also, “non-variceal” was not used in the formal search strategy as it was felt that variceal and the versions of GI bleed would capture any of these articles. An example of the search strategy for MEDLINE is demonstrated in Table 3. A similar strategy will be employed for each database.

**Table 3:** Systematic review search strategy for MEDLINE .tw = title OR abstract; .kw = author-provided keyword; .ti: Title; .kf: author keyword heading word

1	exp Gastrointestinal Hemorrhage/
2	((variceal or GI or gastrointestinal) adj3 (bleed* or hemorrhag* or haemorrhag*)).tw,kf
3	Patient Readmission/
4	(readmiss* or re-admiss* or rehospitalizat* or re-hospitalizat* or readmit* or re-admit* or revisit or re-visit or rehospitalisat* or re-hospitalisat*).ti,kw.
5	1 OR 2
6	3 OR 4
7	5 AND 6

In addition to the electronic search, conference abstracts from North America’s largest meetings in the fields of gastroenterology (American Gastroenterology Association and Canadian Association of Gastroenterology) and emergency medicine (Canadian Association of Emergency Physician (CAEP) and American College of Emergency Physicians (ACEP)) will be reviewed for the most recent five years (2016-21). 

Based on their experience with gastroenterology and emergency medicine, and in discussion with their supervisors, no known experts were identified a priori by the researchers. However, this will be monitored during the abstract review process and if common authors are found, they will be contacted to see if they are aware of any ongoing or unpublished studies. 

Identification of studies

The systematic review will follow the standard Preferred Reporting Items for Systematic Reviews and Meta-Analyses (PRISMA) guidelines study identification process [9]. This will involve a two-stage process. The first will consist of a review of the abstracts identified by the search. Subsequently, the full texts of the abstracts selected as potentially meeting inclusion criteria will be reviewed to determine the final included studies. Each review will be conducted by two researchers (SK, CD) independently and any disagreements will be resolved by discussion. In the case that consensus is unable to be reached via discussion, a third researcher (LB) will review the study to make the final decision. The initial agreement will be quantified for each review round utilizing Cohen’s kappa. Reviewers will utilize Covidence to facilitate the screening process (Covidence systematic review software, Veritas Health Innovation, Melbourne, Australia).

Abstract Review

The initial review round will be to capture any studies remotely relevant to the research question and eliminate any clearly unrelated studies. To proceed to the second round, an abstract must: (i) be of an original research (see note below if reviews are identified in the search strategy); (ii) report data on GI bleeds, or GI-related complications; (iii) not be animal-based research.

To be clear, in this round the researchers will err on the side of inclusion. If an article’s abstract does not clearly state it meets or does not meet a requirement but seems related to the overall theme (e.g., GI bleeds or GI disorders), it will be selected to proceed to the full-text review. 

In the case that a literature review (e.g., systematic, scoping, or narrative) is identified by the search strategy and seems applicable to the research question, the researchers will read the review to determine if any cited studies that are not identified by the search strategy are potentially relevant to include. 

The aim of the second review round will be to identify which studies meet all inclusion criteria. Researchers will utilize a standardized screening tool, adopted from Elzinga et al., to determine the inclusion eligibility of every study [10]. The tool consists of four questions, each addressing a specific inclusion/exclusion criterion.

**Figure 1:**
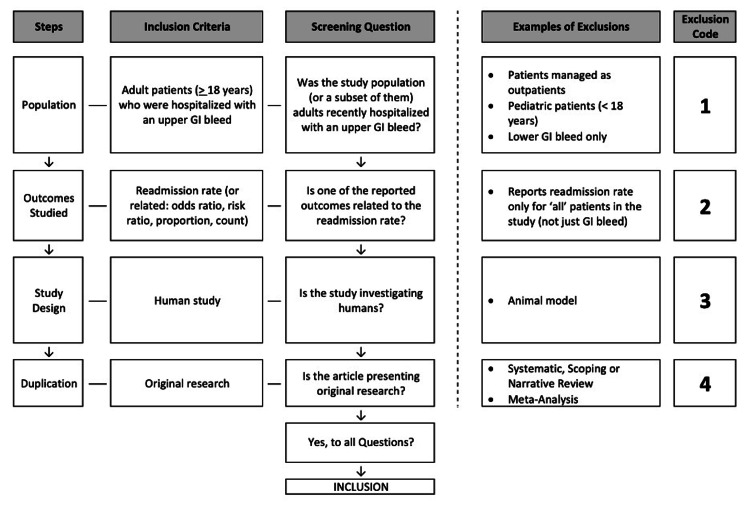
Overview of a standardized full-text screening tool Adapted from: Elzinga et al. (2020) [10]

The first question addresses the appropriate population and whether the study discusses adult (> 18 years old) patients who were hospitalized with a GI bleed. Articles investigating outpatients, pediatric populations, or only lower GI bleeds will be excluded. The second focuses on the outcome and asks whether the readmission rate is reported. Various forms of this (e.g., counts, proportions, ratios) will be accepted if the necessary data is available to combine them. One notable exclusion criterion will be studies that report readmission rates from all pathologies, not just GI bleeds. The third and fourth questions focus on methodology. The studies need to be original, human research (e.g., not animal or a review). Figure 1 displays the screening tool that will be utilized in a sequential manner by researchers. 

## Results

Data Extraction and Synthesis

Data extraction and quality assessment will be undertaken in duplicate. Both researchers (SK, CD) will perform tasks independently, and then compare results. Any disagreements will be resolved by discussion. In the case that consensus is unable to be reached via discussion, a third researcher (LB) will review the article to make the final decision. 

Data Extraction

A standardized data extraction tool will be utilized for all studies. Data will be extracted into Microsoft Excel (Microsoft Corporation, Redmond, Washington, USA). This is included in the Appendix. In general, it will incorporate four themes (study characteristics, patient characteristics, outcomes, and quality). Table 4 summarizes the four themes and sample questions within each.

**Table 4:** Data extraction core themes and sample data points LOS: length of stay

Theme	Sample variables
Study characteristics	Publication date; Corresponding author; Publication journal; Study methodology (e.g., trial, cohort, case-control, prospective or not, etc); Cohort, case, or population definition; Study geographic location; Study time frame
Patient characteristics	Sample size; Baseline characteristics (age, comorbidities, sex, ethnicity, medications the patient was receiving prior to admission); Anticoagulation status; Etiology of GI bleed, lesion on endoscopy; Treatment received at the index visit; Hospitalization LOS for the index visit; GI bleed severity (e.g., Rockall score or Glasgow Blatchford score components)
Outcomes	Readmission rate (all-cause and GI bleed-specific), Mortality (all-cause and GI bleed-specific), Subsequent hospitalization LOS Readmission diagnosis
Quality (Downs & Black, 1998) [11]	Reporting; External Validity; Internal Validity - Bias; Internal Validity - Confounding; Power

Missing data will be handled by contacting the corresponding author requesting the information. All authors will be contacted only once by email and have 30 days to respond. After this period, articles will be removed from the analysis.

Quality Assessment

Quality assessment will be conducted utilizing the Downs and Black Quality Assessment Scale [11]. This method was chosen due to its applicability to both randomized and non-randomized studies.

Data analysis

Summary measures for each included study and patient baseline characteristics will be reported along with associated 95% confidence intervals. For the entire population, the incidence of all-cause readmission will be pooled using a random-effects model, along with secondary outcomes (e.g., GI-specific readmission, mortality, readmission diagnosis, and length of stay), if feasible based on heterogeneity between studies and information reported in each included study. 

In the situation where different timeframes are used to measure outcomes (e.g., seven-day versus 30-day readmission rate), there will be a check for a linear effect performed to see if outcomes can be extrapolated and combined to a similar time reference. 

The degree of heterogeneity will be formally assessed by calculating I^2^. 

Stratified analyses will also be conducted for various groups, including: Randomized control trials (RCTs) versus observational studies, interventional versus epidemiological studies, low versus high-risk UGIB (based on clinical scoring scales: Rockall > 1, Glasgow-Blatchford scores (GBS) > 0), high and low-quality studies, and time period. For specific subgroups, odds ratios (and 95% confidence interval) will also be calculated using a random-effects model to determine their overall impact on readmission (e.g., age, sex, degree of comorbidities, variceal, index treatment, and anticoagulation status). 

## Discussion

Although there have been advancements in treatment for UGIB, the mortality rate has remained relatively unchanged over the last 50 years [3]. Advances in endoscopic therapy and prevention of UGIB lesions, through treatment of *Helicobacter pylori* infection; use of proton pump inhibitors (PPIs); and use of selective cyclooxygenase-2 (COX-2) inhibitors, have affected incidence rates and morbidity. Conversely, risk factors of UGIB, including advancing age, renal failure, and exposure to antiplatelet therapy or systemic anticoagulation, have increased [12,13]. It is crucial to reassess the impact of UGIB on various outcomes because of changing epidemiology. 

GI bleeding can be fatal and is one of the leading causes of readmission to hospital [3]. For certain diseases such as congestive heart failure, acute myocardial infarction, and pneumonia, 30-day hospital readmission is now a quality indicator [14]. Readmission within 30 days after the first episode of bleeding poses a significant burden on the healthcare system [15]. One study reported a 13% 30-day all-cause readmission rate, frequently due to recurrent bleeding [16]. It is seen that 30-day readmission led to an increase in health resource utilization, morbidity, and a double-fold increase in-hospital mortality when compared to patients who did not require readmission [16]. While in-hospital and 30-day mortality have been well studied in UGIB, there is limited data on 30-day all-cause hospital readmission of patients after discharge from hospital for a primary UGIB episode [14]. This study aims to address this important evidence gap. 

## Conclusions

This report describes the systematic review and meta-analysis protocol that will be utilized to determine the readmission rate of UGIB. It will highlight this for the general population, as well as certain high-risk subgroups. By addressing this current evidence gap, practitioners will be able to identify which patients are at greatest risk of decompensation post-discharge and this can inform future studies evaluating potential interventions to mitigate this risk.

## Appendices

Table 5 is the data extraction tool that will be utilized by reviewers.

**Table 5:** Standardized data extraction tool GBS: Glasgow-Blatchford Bleeding Score; LOS: length of stay; NSAID: non-steroidal anti-inflammatory drugs; TIPS: transjugular intrahepatic portosystemic shunt; UGIB: upper gastrointestinal bleed; DOI: digital object identifier; SSRIs: selective serotonin reuptake inhibitors; COX-2: cyclooxygenase-2.

Study characteristics	Title
	First author
	Corresponding author
	Contact information for the corresponding author
	DOI
	Citation
	Journal name (or location identified)
	State/province, country of a study conducted
	Study design/methodology
	Cohort, case, or population definition
	For trials: intervention and comparison definition
	Site status: single-center, multi-center
Patient characteristics	Total included patients with GI bleed (n)
	For trials: number in intervention and comparison arm
	Age
	Sex
	Ethnicity
	Treatment at index visit (select all that apply): endoscopy, banding, epinephrine injection, proton pump inhibitor, a somatostatin analog, antimicrobial therapy, TIPS, blood transfusion, angiography/embolization, H. pylori treatment
	Index etiology of GI bleed
	Index lesion(s) visualized on endoscopy
	Index severity of UGIB (e.g., Rockall or GBS score*)
	Index hospitalization LOS*
	Index ICU LOS
	Variceal (yes/no)
	Non-variceal (yes/no)
	Comorbidities [as defined by the study, e.g., count, Charlson index, Elixhauser index]
	Comorbidities list
	Presence of renal failure (yes/no; study definition)
	Presence of liver failure (yes/no; study definition)
	Presence of malignancy
	Presence of heart failure
	History of GI bleed before index visit (yes/no)
	Anticoagulation status (yes/no)
	Anticoagulation therapy (medication and dose if known)
	History of significant NSAID use (yes/no; study definition)
	Other medication history reported by study (e.g., bisphosphonates, SSRIs, COX-2 inhibitors)
	If in a non-socialized country, insurance status
	Re-presentation severity of UGIB (e.g., Rockall or GBS score)
	Re-presentation (ED*) heart rate (GBS component)
	Re-presentation (ED) blood pressure (GBS component)
	Re-presentation hemoglobin (GBS component)
	Re-presentation etiology of GI bleed (GBS component)
	Re-presentation syncope occurred (GBS component)
	Re-presentation melena present (GBS component)
	Re-presentation lesion(s) visualized on endoscopy (including degree noticed)
Outcome All outcome variables will be captured for the total population, as well as any subgroup categories that will permit pooled analysis of subgroups later For all variables, the statistic, measure of spread, and significant level will be recorded	Readmission rate (all-cause) (and time definition, e.g. 30-day)
	Readmission rate (GI bleed) (and time definition, e.g. 30-day)
	Mortality (all-cause) (and time definition, e.g. 30-day)
	Mortality (GI bleed) (and time definition, e.g. 30-day)
	Subsequent hospitalization LOS
	Subsequent hospitalization ICU LOS
	Shock on subsequent presentation (yes/no)
	Definition of shock in study
	Readmission diagnosis
	ED visit without admission
Quality (24)	Is the hypothesis/aim/objective of the study clearly described? (yes/no/uncertain)
	Are the main outcomes to be measured clearly described in the Introduction or Methods section? (yes/no/uncertain)
	Are the characteristics of the patients included in the study clearly described? (yes/no/uncertain)
	Are the interventions of interest clearly described? (yes/no/uncertain)
	Are the distributions of principal confounders in each group of subjects to be compared clearly described? Are the main findings of the study clearly described? (yes/no/uncertain)
	Does the study provide estimates of the random variability in the data for the main outcomes? (yes/no/uncertain)
	Have all important adverse events that may be a consequence of the intervention been reported? (yes/no/uncertain)
	Have the characteristics of patients lost to follow-up been described? (yes/no/uncertain)
	Have actual probability values been reported (e.g. 0.035 rather than <0.05) for the main outcomes except where the probability value is less than 0.001? (yes/no/uncertain)
	Were the subjects asked to participate in the study representative of the entire population from which they were recruited? (yes/no/uncertain)
	Were those subjects who were prepared to participate representative of the entire population from which they were recruited? (yes/no/uncertain)
	Were the staff, places, and facilities where the patients were treated, representative of the treatment the majority of patients receive? (yes/no/uncertain)
	Was an attempt made to blind those measuring the main outcomes of the intervention? (yes/no/uncertain)
	If any of the results of the study were based on “data dredging”, was this made clear? (yes/no/uncertain)
	In trials and cohort studies, do the analyses adjust for divergent lengths of follow-up of patients, or in case-control studies, is the time period between the intervention and outcome the same for cases and controls? (yes/no/uncertain)
	Were the statistical tests used to assess the main outcomes appropriate? (yes/no/uncertain)
	Was compliance with the intervention/s reliable? (yes/no/uncertain)
	Were the main outcome measures used accurate (valid and reliable)? (yes/no/uncertain)
	Were the patients in divergent intervention groups (trials and cohort studies) or were the cases and controls (case-control studies) recruited from the same population? (yes/no/uncertain)
	Were study subjects in divergent intervention groups (trials and cohort studies) or were the cases and controls (case-control studies) recruited over the same period of time? (yes/no/uncertain)
	Were study subjects randomized to intervention groups? (yes/no/uncertain)
	Was the randomized intervention assignment concealed from both patients and health care staff until recruitment was complete and irrevocable? (yes/no/uncertain)
	Was there an adequate adjustment for confounding in the analyses from which the main findings were drawn? (yes/no/uncertain)
	Were losses of patients to follow-up taken into account? (yes/no/uncertain)
	Did the study have sufficient power to detect a clinically important effect where the probability value for a divergence being due to chance is less than 5%? (yes/no/uncertain)
